# Phylogenetic analyses of the mitochondrial, plastid, and nuclear genes of *Babesia* sp. Mymensingh and its naming as *Babesia naoakii* n. sp.

**DOI:** 10.1186/s13071-022-05374-9

**Published:** 2022-08-24

**Authors:** Thillaiampalam Sivakumar, Bumduuren Tuvshintulga, Davaajav Otgonsuren, Enkhbaatar Batmagnai, Believe Ahedor, Hemal Kothalawala, Singarayar Caniciyas Vimalakumar, Seekkuge Susil Priyantha Silva, Junya Yamagishi, Naoaki Yokoyama

**Affiliations:** 1 National Research Center for Protozoan Diseases, Obihiro University of Agriculture and Veterinary Medicine, Inada-cho, Obihiro, Hokkaido 080-8555 Japan; 2 Veterinary Research Institute, Peradeniya, Sri Lanka; 3 Department of Animal Production and Health, Deputy Director’s Office, Mannar, Sri Lanka; 4 Department of Animal Production and Health, Peradeniya, Sri Lanka; 5 Division of Collaboration and Education, International Institute for Zoonosis Control, Hokkaido University, Sapporo, Hokkaido Japan; 6 OIE Reference Laboratory for Bovine Babesiosis, National Research Center for Protozoan Diseases, Obihiro University of Agriculture and Veterinary Medicine, Obihiro, Hokkaido Japan

**Keywords:** *Babesia* sp. Mymensingh, *Babesia naoakii* n. sp., Cattle, Novel species, Phylogeny

## Abstract

**Background:**

The recently discovered *Babesia* sp. Mymensingh, which causes clinical bovine babesiosis, has a wide geographical distribution. We investigated the phylogenetic position of *Babesia* sp. Mymensingh using its mitochondrial, plastid, and nuclear genes. Based on morphological and molecular data, *Babesia* sp. Mymensingh is a unique species and we named it as *Babesia naoakii* n. sp.

**Methods:**

A blood DNA sample from a *Babesia* sp. Mymensingh-infected cow was subjected to genome sequencing to obtain the sequences of mitochondrial, plastid, and nuclear genes. Six phylogenetic trees were then constructed with (1) concatenated amino acid sequences of cytochrome oxidase subunit I, cytochrome oxidase subunit III, and cytochrome b genes of the mitochondrial genome; (2) 16S rRNA of the plastid genome; (3) nucleotide sequences of the elongation factor Tu gene of the plastid genome; (4) ITS1-5.8S rRNA-ITS2; (5) concatenated nucleotide sequences of 89 nuclear genes; and (6) concatenated amino acid sequences translated from the 89 nuclear genes.

**Results:**

In all six phylogenetic trees, *B. naoakii* n. sp. formed a sister clade to the common ancestor of *Babesia bigemina* and *B. ovata*. The concatenated nuclear genes of *B. naoakii* n. sp. and their translated amino acid sequences shared lower identity scores with the sequences from *B. bigemina* (82.7% and 84.7%, respectively) and *B. ovata* (83.5% and 85.5%, respectively) compared with the identity scores shared between the *B. bigemina* and *B. ovata* sequences (86.3% and 87.9%, respectively).

**Conclusions:**

Our study showed that *B. naoakii* n. sp. occupies a unique phylogenetic position distinct from existing *Babesia* species. Our findings, together with morphological differences, identify *B. naoakii* n. sp. as a distinct parasite species.

**Graphical Abstract:**

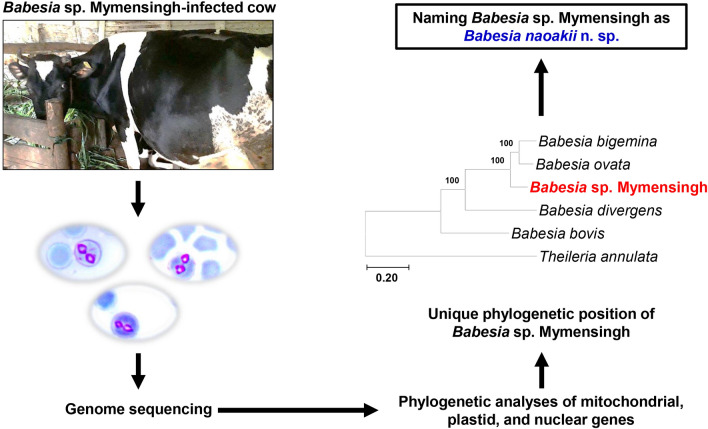

**Supplementary Information:**

The online version contains supplementary material available at 10.1186/s13071-022-05374-9.

## Background

*Babesia* species are intraerythrocytic protozoan parasites that are transmitted to vertebrate hosts via tick vectors [1]. *Babesia* parasites invade, asexually reproduce in, and egress from red blood cells in vertebrate hosts, resulting in intravascular haemolytic anaemia [2]. As a result, babesiosis leads to a clinically serious condition in infected animals. In cattle, *Babesia* parasite species cause bovine babesiosis, which is a fatal disease that mostly affects cattle in tropical and subtropical regions of the world [3]. The disease results in huge economic losses to the cattle industry due to loss of production, cost of treatment and prevention, mortality, and restrictions to international cattle movement. Several species of *Babesia*, such as *Babesia bovis*, *B. bigemina*, *B. divergens*, *B. ovata*, *B. major*, and *B. occultans*, infect cattle, but only *B. bovis*, *B. bigemina*, and *B. divergens* are known to cause clinical babesiosis [3].

However, our recent investigation found that *Babesia* sp. Mymensingh, which was first characterised based on an 18S ribosomal RNA (18S rRNA) sequence in a cow in Bangladesh [4], can cause clinical babesiosis in cattle [5]. Additional studies detected *Babesia* sp. Mymensingh in cattle, buffalo, sheep, goats, and camels in Asian, African, and American countries [6–8]. Despite the clinical significance and wide geographical distribution, the taxonomy of *Babesia* sp. Mymensingh is still incomplete, because a scientific name for this parasite species has not been assigned yet. Correct taxonomic description is vital for the smooth communication of biological information. Therefore, the incomplete taxonomy of *Babesia* sp. Mymensingh might create unnecessary confusion. For example, a recent survey detected an 18S rRNA gene closely related to that of *Babesia* sp. Mymensingh in cattle, but the authors described the organism as *B. bigemina* [9].

**Fig. 1:**
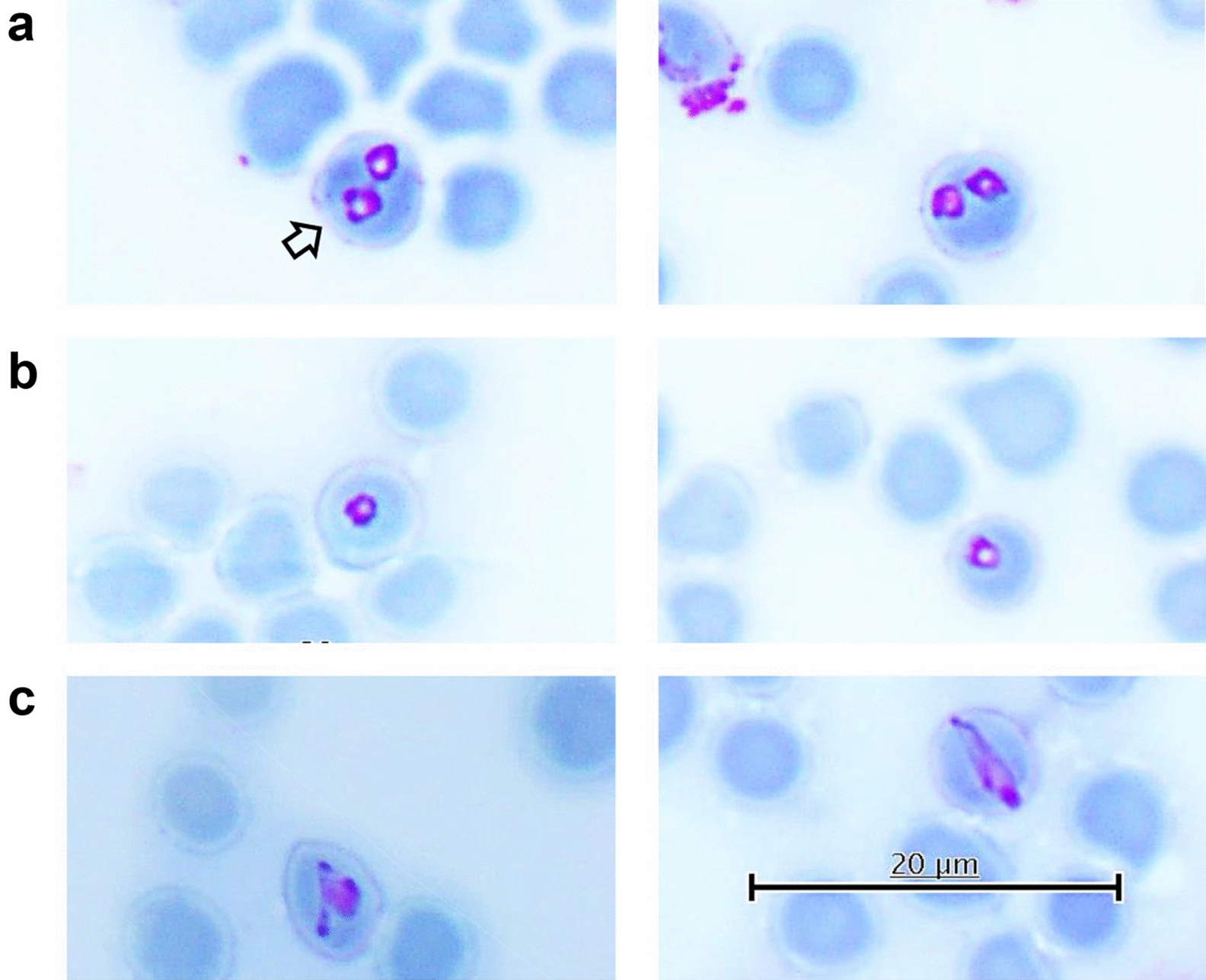
Micrographs of *B. naoakii* n. sp. Microscopic examination of a Giemsa-stained thin blood smear (type-material) from an infected cow revealed the presence of different morphological forms, including paired pyriforms (**a**), ring forms (**b**), and irregularly shaped single forms (**c**), within red blood cells. The holotype is marked with an arrow in **a**

Morphological distinctions demonstrate that *Babesia* sp. Mymensingh is a separate parasite species (Fig. 1), but its phylogenetic position is unclear [5]. Sequences of only three genes from *Babesia* sp. Mymensingh, comprising 18S rRNA, cytochrome oxidase subunit III (*cox3*), and apical membrane antigen 1 (*ama-1*), exist in the databases. In phylogenetic trees, the 18S rRNA gene and *cox3* of *Babesia* sp. Mymensingh form sister clades to those of *B. bigemina* [5]. In contrast, *Babesia* sp. Mymensingh *ama-1* sequences form a sister clade to the common ancestor of *B. bigemina* and *B. ovata* [5–7]. Because of these discrepancies, the phylogenetic position of *Babesia* sp. Mymensingh is still undetermined.

Apicomplexan parasites, including *Babesia* species, possess three genomes: mitochondrial, plastid, and nuclear genomes. Previous studies found that phylogenetic analyses of genes from all three genomes are useful for identifying congeneric apicomplexan species. The mitochondrial and plastid genes are considered taxonomic markers of closely related species due to their low rates of recombination, relatively rapid rates of evolution, and high phylogenetic signal indexes [10, 11]. In particular, concatenated genes of the mitochondrial genome and 16S ribosomal RNA (16S rRNA) and elongation factor Tu (*tufA*) of plastid genomes have been widely used for phylogenetic analyses [12, 13]. In addition, phylogenies based on concatenated sequences of nuclear genes have also been widely employed to aid the discovery of novel parasite species [14]. In the present study, therefore, we analysed the concatenated mitochondrial genes, 16S rRNA and *tufA* of the plastid genome, and concatenated nuclear genes and their translated amino acid sequences to determine the phylogenetic position of *Babesia* sp. Mymensingh. We also investigated whether *Babesia* sp. Mymensingh forms a distinct clade in a phylogenetic tree constructed using the internal transcribed spacer 1–5.8S ribosomal RNA-internal transcribed spacer 2 (ITS1-5.8S rRNA-ITS2) region, which has relatively high diversity within a given parasite population compared to other marker genes [15].

## Methods

To obtain gene sequences for phylogenetic analyses, the genome of *Babesia* sp. Mymensingh was sequenced. In brief, 10 ml of whole blood was drawn from a cow infected with *Babesia* sp. Mymensingh in the Badulla district of Sri Lanka in 2017 using EDTA as an anticoagulant [5]. Polymerase chain reaction (PCR) and sequencing analyses detected only *Babesia* sp. Mymensingh, not any other *Babesia* species [5]. The parasitaemia, as determined by the microscopic examination of a Giemsa-stained thin blood smear, was 2.3%. A Plasmodipur filter (EuroProxima, Arnhem, Netherlands) was used to remove leukocytes from the collected blood. The DNA was then extracted using the QIAamp DNA Blood Maxi Kit (Qiagen, Valencia, CA, USA) following the manufacturer’s instructions.

Subsequently, the DNA sample was subjected to whole-genome amplification using the GenomiPhi V2 DNA Amplification Kit (GE Healthcare, Chicago, IL, USA) following the manufacturer’s instructions. A library was constructed with the Nextera XT DNA Library Prep Kit (Illumina, San Diego, CA, USA) and then subjected to 300-bp paired-end sequencing with MiSeq (Illumina). The quality of the reads was controlled with Trimmomatic [16] using the following parameters: LEADING:20, TRAILING:20, SLIDINGWINDOW:4:15, and MINLEN:100. The trimmed reads, which were 1,922,738,761 bp in total, were then assembled with paired-end ABySS [17] using *k *= 64. The de novo assembled sequence was 13.35 Mbp and consisted of 90,524 contigs. Potential open reading frames and amino acid sequences coded by each contig were specified using getorf in the European Molecular Biology Open Software Suite (EMBOSS) [18]. Amino acid sequences and coding sequences for *B. bovis*, *B. bigemina*, *B. ovata*, *B. divergens*, and *Theileria annulata* were retrieved from PiroplasmaDB (https://piroplasmadb.org). Genes homologous to those of *Babesia* sp. Mymensingh were identified using Blastp, and the corresponding amino acid sequences and coding sequences were then obtained. The *Babesia* sp. Mymensingh sequences used in this study were registered with the DNA Data Bank of Japan (DDBJ) under Accession numbers LC684678-LC684772.

The following sequences from *Babesia* sp. Mymensingh, together with homologous sequences from other *Babesia* species, were used to construct six phylogenetic trees: (1) concatenated amino acid sequences of cytochrome oxidase subunit I (*cox1*), *cox3*, and cytochrome b (*cob*) genes of the mitochondrial genome; (2) 16S rRNA of the plastid genome; (3) nucleotide sequences of *tufA* gene of the plastid genome; (4) ITS1-5.8S rRNA-ITS2; (5) concatenated nucleotide sequences of 89 nuclear genes (Additional file 1: Table S1); and (6) concatenated amino acid sequences translated from the nuclear genes. The 89 nuclear-encoded gene sequences had lengths comparable to those of other bovine *Babesia* species, including *B. bovis* (T2Bo strain) [19], *B. bigemina* (BBOND strain) [20], *B. ovata* (Miyake strain) [21], *B. divergens* (Rouen 1987 strain) [22], and *T. annulata* (Ankara strain) [23]. The *Babesia* sp. Mymensingh sequences and those of other parasite species were processed for multiple alignment using fast Fourier transform software (https://mafft.cbrc.jp/alignment/server/) [24]. The alignments of 16S rRNA, *tufA*, ITS1-5.8S rRNA-ITS2, and concatenated nuclear genes consisted of 877, 934, 421, and 103,646 nucleotides, respectively. The alignment of the concatenated amino acid sequences translated from the mitochondrial and nuclear genes consisted of 986 and 34,846 residues, respectively. The alignments were analysed with Molecular Evolutionary Genetics Analysis, version X, software (MEGA X) [25], and best-fitting substitution models were predicted based on the lowest Akaike information criterion value. Maximum likelihood phylogenetic trees rooted with either *Plasmodium falciparum*, *Toxoplasma gondii*, or *T. annulata* sequences were then constructed based on the general time reversible model (nucleotide sequences) [26], the Le–Gascuel 2008 model (concatenated amino acid sequences of nuclear genes) [27], and the general reversible mitochondrial model (concatenated amino acid sequences of mitochondrial genes) [28] using MEGA X.

The concatenated nuclear genes of *Babesia* sp. Mymensingh, *B. bigemina*, and *B. ovata* were analysed for single-nucleotide polymorphisms (SNPs) using DNA Sequence Polymorphism software [29], as well as for nucleotide and amino acid identity scores using EMBOSS Stretcher (https://www.ebi.ac.uk/Tools/psa/emboss_stretcher/).

## Results

Order Piroplasmida Wenyon, 1926

Family Babesiidae Poche, 1913

Genus *Babesia* Starcovici, 1893

### *Babesia naoakii* n. sp.

Type host: Cattle (*Bos taurus*).

Other hosts: The parasite’s DNA has been detected in water buffalo, goats, sheep, and Bactrian camels.

Type locality: Badulla district (6°59ʹ31.2″N, 81°03ʹ00.3″E), Sri Lanka.

Other localities: Bangladesh [4], India [9], Mongolia [7, 8], Vietnam [6], Uganda [6], and Argentina [6]

Type material: A blood DNA sample and a stained thin blood smear containing the holotype (Fig. 1) prepared from an infected cow have been registered with the Material Management Center, Ministry of Education, Culture, Sports, Science, and Technology, Japan with the Accession number OUMR-2021-00077 and OUMR-2022-00010, respectively.

Vector: Unknown.

Representative DNA sequences: Gene sequences with the following Accession numbers have been submitted to DDBJ: LC684678-LC684772.

ZooBank registration: To comply with the regulations set out in article 8.5 of the amended 2012 version of the International Code of Zoological Nomenclature (ICZN) [30], details of the new species have been submitted to ZooBank. The Life Science Identifier (LSID) of the article is urn:lsid:zoobank.org:pub:4DBE1F9C-4F97-4655-89F8-CF1DEE1BEC9C. The LSID for the new name *Babesia naoakii* n. sp. is urn:lsid:zoobank.org:act:842EB419-FBB5-4C1B-ABF8-74DC947A02BE.

Description: Microscopically, *B. naoakii* n. sp. appears as paired pyriforms, elongated or irregularly shaped single forms, and ring forms within infected erythrocytes [5]. The paired pyriforms form an obtuse angle. The length and width of the paired pyriforms are 2.25 μm to 3.04 μm and 1.58 μm to 2.20 μm, respectively, while the ring forms are 1.52 μm to 1.97 μm in diameter [5]. A PCR assay using a forward (TGGCGCCGACTTCCTGGAGCCCATCTCCAA) and reverse (AGCTGGGGCCCTCCTTCGATGAACCGTCGG) primer targeting the *ama-1* gene specifically detects *B. naoakii* n. sp. infection in host animals [5].

Etymology: *Babesia naoakii* n. sp. was named after Naoaki Yokoyama, who first observed this species under a microscope and identified the morphological distinctions.

### Molecular phylogeny

**Fig. 2:**
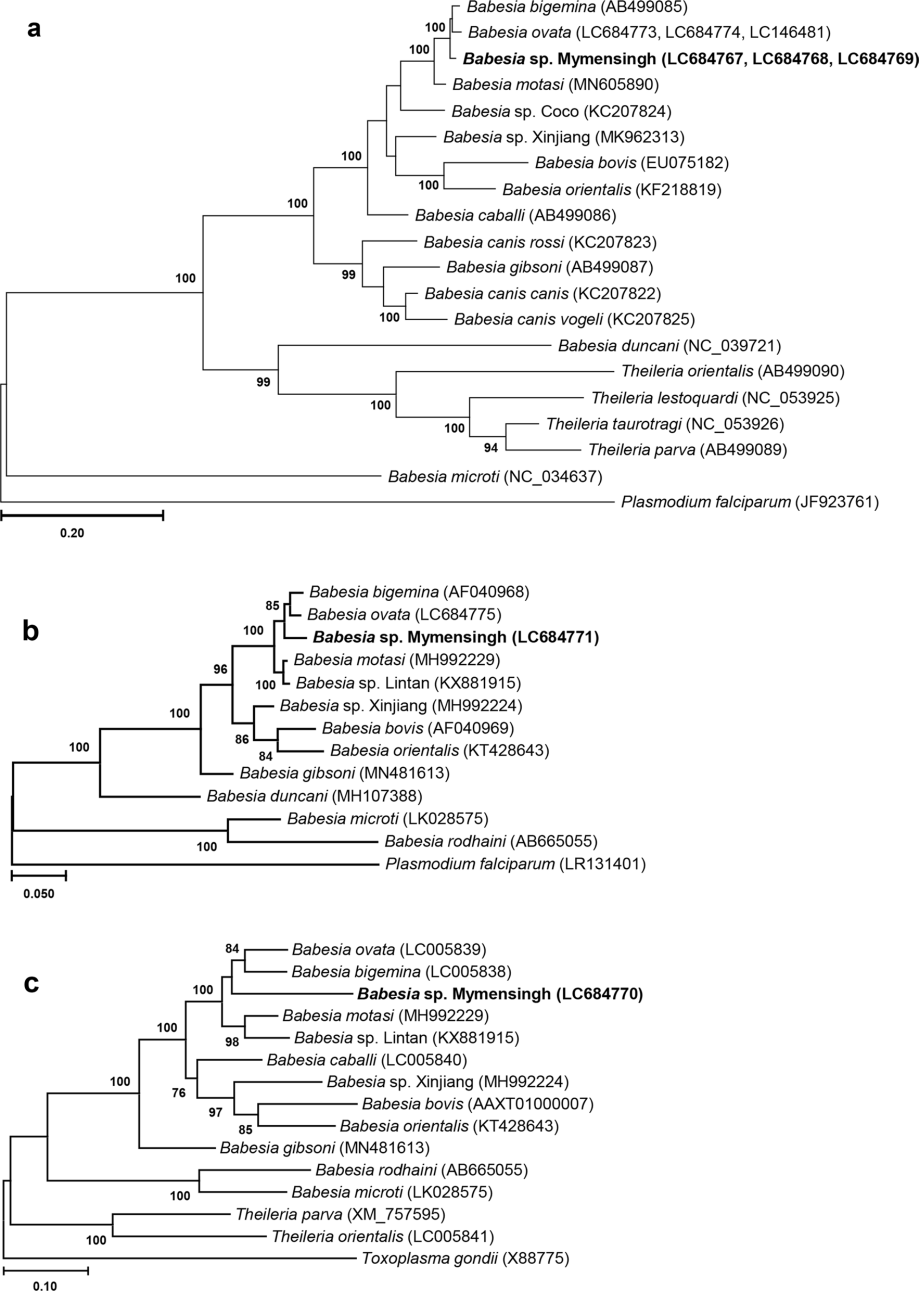
Phylogenetic analyses of mitochondrial and plastid genes. Maximum likelihood phylogenetic trees were constructed with concatenated amino acid sequences of cytochrome oxidase subunit I (*cox1*), *cox3*, and cytochrome b genes of the mitochondrial genome (**a**), and the nucleotide sequences of 16S rRNA (**b**) and *tufa* genes (**c**) of the plastid genome. In all three phylogenetic trees, *Babesia naoakii* n. sp. formed a sister clade to the common ancestor of *B. bigemina* and *B. ovata*

**Fig. 3:**
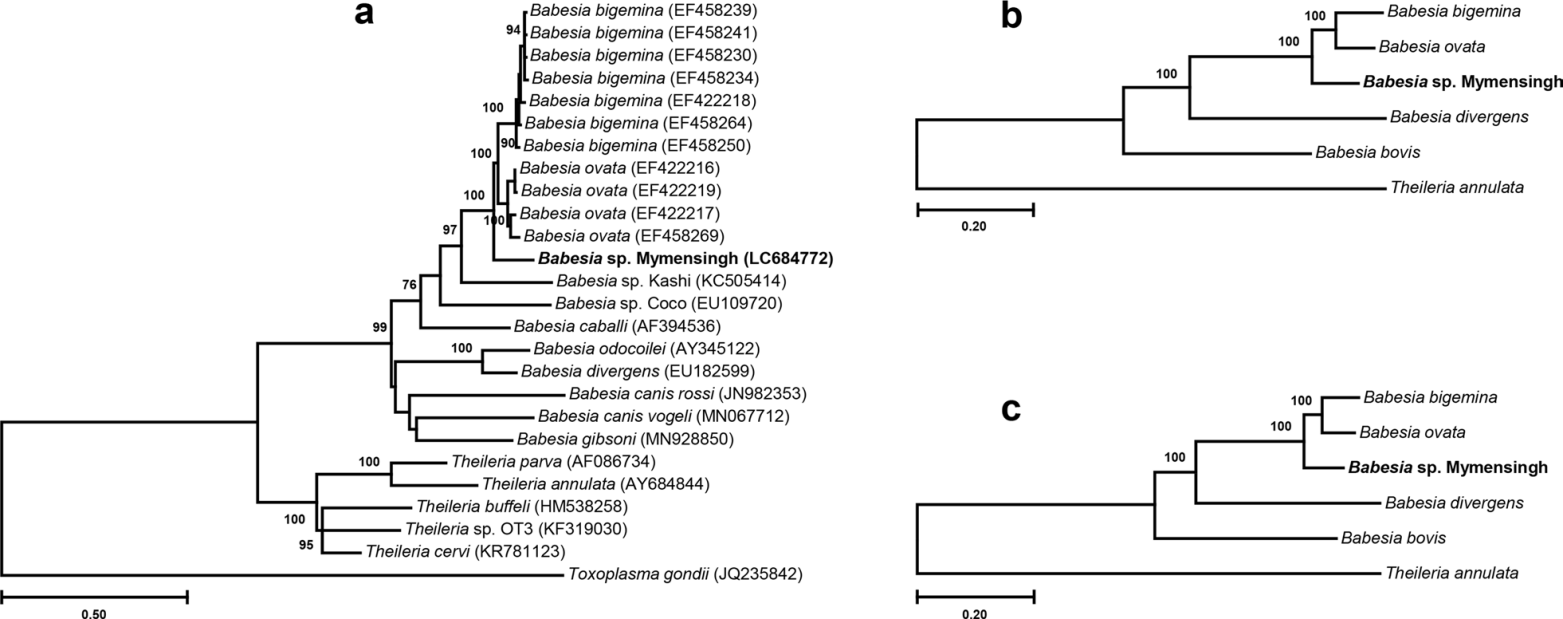
Phylogenetic analyses of ITS1-5.8S rRNA-ITS2 and concatenated nuclear genes. Maximum likelihood phylogenetic trees were constructed with ITS1-5.8S rRNA-ITS2 (**a**), concatenated nucleotide sequences encoding 89 nuclear genes (**b**), and concatenated amino acid sequences translated from 89 nuclear genes (**c**). In all three phylogenetic trees, *Babesia naoakii* n. sp. formed a sister clade to the common ancestor of *B. bigemina* and *B. ovata*

In all six phylogenetic trees, *B. naoakii* n. sp. formed a sister clade to the common ancestor of *B. ovata* and *B. bigemina* (Figs. 2 and 3). The position of *Babesia* sp. Mymensingh was further confirmed in additional phylogenetic trees constructed with concatenated nucleotide sequences of nuclear genes (*n* = 30; Additional file 2: Table S2) and their translated amino acid sequences from *B. naoakii* n. sp., *B. bovis* [19], *B. bigemina* [20], *B. ovata* [21], *B. divergens* [22], *Babesia* sp. Xinjiang [31], *T. annulata* [23], *Theileria parva* [32], *Theileria orientalis* [33], and *P. falciparum* [34] (Additional file 3: Fig. S1). Apart from the 16S rRNA and *tufA* phylogenies (Fig. 2b and c), the separation of *B. naoakii* n. sp. was supported by high bootstrap values (Figs. 2a and 3). A reason for the relatively low bootstrap values in the 16S rRNA gene and *tufA* phylogenies could be the partial fragments used in their construction. Additionally, our sequencing analyses found more SNPs between *B. naoakii* n. sp. and *B. bigemina* and between *B. naoakii* n. sp. and *B. ovata* than between *B. bigemina* and *B. ovata* (Table 1). Similarly, the nucleotide and amino acid identity scores between *B. naoakii* n. sp. and *B. bigemina* and between *B. naoakii* n. sp. and *B. ovata* were lower than those between *B. bigemina* and *B. ovata* (Table 1).

**Table 1:** Single-nucleotide polymorphisms (SNPs) and nucleotide and amino acid identity scores shared among concatenated nuclear genes (*n *= 89) from *Babesia bigemina*, *B. ovata*, and *Babesia naoakii* n. sp.

Parameter	*B. bigemina* vs *B. ovata*	*Babesia naoakii* n. sp. vs *B. bigemina*	*Babesia naoakii* n. sp. vs *B. ovata*
SNPs	14,513	18,580	17,538
Nucleotide identity	86.3%	82.7%	83.5%
Amino acid identity	87.9%	84.7%	85.5%

## Discussion

Taken together, our present findings on the phylogenetic positions, SNPs, and sequence identity scores indicate that *B. naoakii* n. sp. is a distinct *Babesia* species.

*Babesia naoakii* n. sp. formed a sister clade to the common ancestor of *B. ovata* and *B. bigemina*. *Babesia bigemina* and *B. ovata* are two distinct *Babesia* species: *B. bigemina* is a virulent species capable of causing clinical babesiosis in cattle [3], whereas *B. ovata* is a relatively benign species that may cause clinical babesiosis in immunocompromised or *T. orientalis*-infected cattle [35, 36]. *Babesia bigemina* is transmitted by *Rhipicephalus* (*Boophilus*) ticks, while *B. ovata* is transmitted by *Haemaphysalis* ticks [3]. The distribution of *B. bigemina* and *B. ovata* also differs: *B. bigemina* has a worldwide distribution, whereas *B. ovata* is limited to a few Asian countries [3, 37]. In addition, recent genomic analyses have demonstrated that these two parasite species differ genetically from each other [21]. Therefore, the finding that *B. naoakii* n. sp. forms a sister clade to the common ancestor of two different parasite species, *B. bigemina* and *B. ovata*, convincingly distinguishes it as a distinct *Babesia* species.

Similar to *B. bigemina*, *B. naoakii* n. sp. may apparently cause clinical disease in cattle. For instance, fever, anaemia, and haemoglobinuria were observed in a naturally infected cow from Sri Lanka. The animal red blood cell counts, haemoglobin concentration, and haematocrit values fell below the normal range. Further research is needed to gain more information on the clinical picture of *B. naoakii* n. sp. infection in cattle.

## Conclusion

Based on morphology and data from mitochondrial, plastid, and nuclear genes, *Babesia* sp. Mymensingh is named *B. naoakii* n. sp.

## Supplementary Information

**Additional file 1: Table S1**. List of nuclear genes (*n* = 89) used for concatenated phylogenetic trees in Fig. 3.

**Additional file 2: Table S2**. List of nuclear genes (*n* = 30) used for concatenated phylogenetic trees in Fig. S1.

**Additional file 3: Figure S1**. Phylogenetic trees constructed with concatenated nuclear genes. Nucleotide sequences of 30 nuclear genes (panel **a**) and their translated amino acid sequences (panel **b**) were concatenated and used to construct maximum likelihood phylogenetic trees based on general time reversible and Le–Gascuel 2008 models, respectively. In both phylogenetic trees, *Babesia naoakii* n. sp. formed a sister clade to the common ancestor of *B. bigemina* and *B. ovata*.

## Data Availability

All data generated or analysed during this study are included in this published article and its Additional files.

## Abbreviations

*ama-1*: Apical membrane antigen 1
*cob*: Cytochrome b
*cox1*: Cytochrome oxidase subunit I
*cox3*: Cytochrome oxidase subunit III
DDBJ: DNA Data Bank of Japan
EMBOSS: European Molecular Biology Open Software Suite
ITS1-5.8S rRNA-ITS2: Internal transcribed spacer 1–5.8S ribosomal RNA-internal transcribed spacer 2
MEGA X: Molecular Evolutionary Genetics Analysis version X software
PCR: Polymerase chain reaction
*tufA*: Elongation factor Tu
16S rRNA: 16S ribosomal RNA
18S rRNA: 18S ribosomal RNA
