# Supplementary figures and images for: Phylogenetic analyses of the mitochondrial, plastid, and nuclear genes of Babesia sp. Mymensingh and its naming as Babesia naoakii n. sp
Source: Parasit Vectors. 2022 Aug 24;15:299. doi: 10.1186/s13071-022-05374-9 (PMC9404586; doi:10.1186/s13071-022-05374-9)

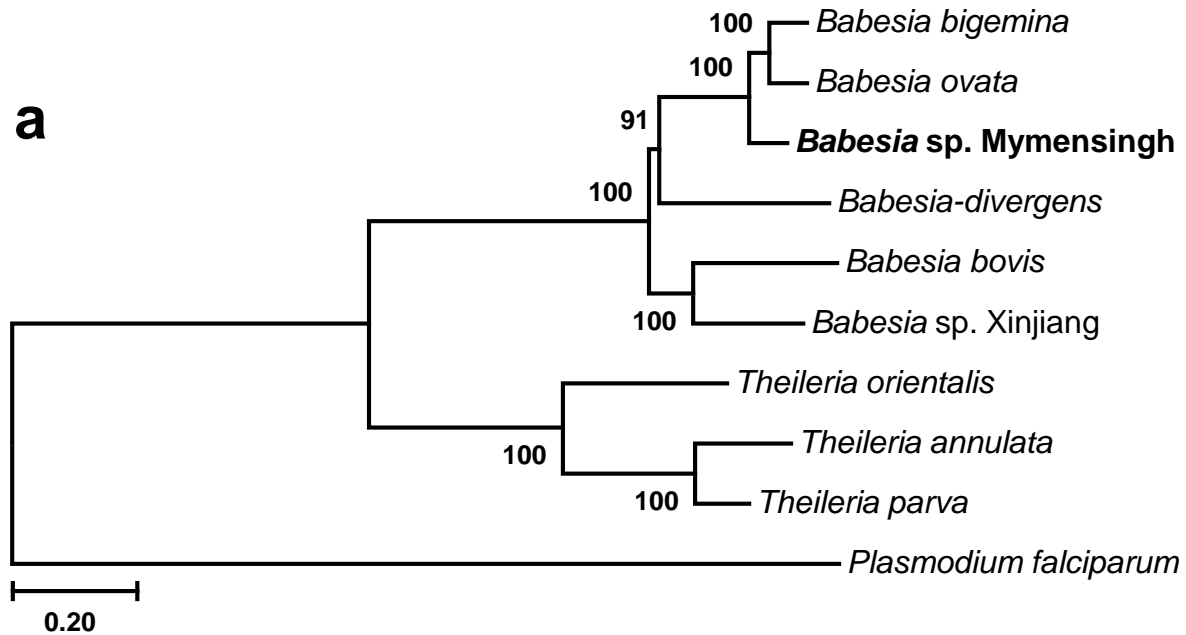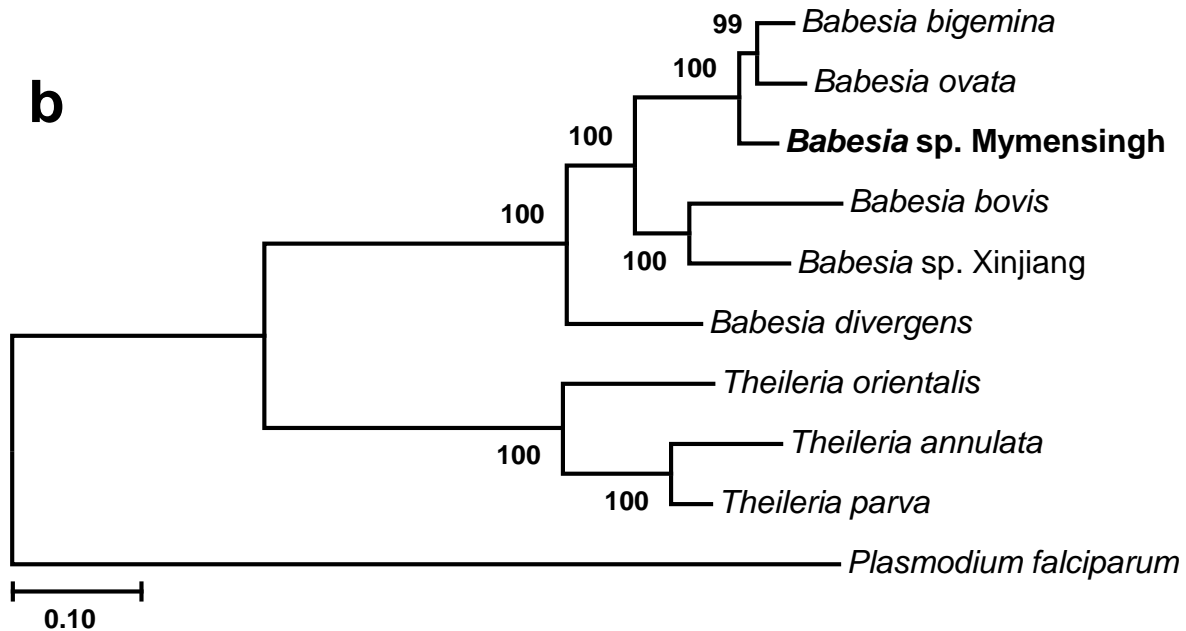

Supplement: Supplementary file 3 — Additional file 3: Figure S1. Phylogenetic trees constructed with concatenated nuclear genes. Nucleotide sequences of 30 nuclear genes (panel a) and their translated amino acid sequences (panel b) were concatenated and used to construct maximum likelihood phylogenetic trees based on general time reversible and Le–Gascuel 2008 models, respectively. In both phylogenetic trees, Babesia naoakii n. sp. formed a sister clade to the common ancestor of B. bigemina and B. ovata. [file 13071_2022_5374_MOESM3_ESM.pdf]
